# OA21 Ankylosing Spondylitis management with Secukinumab but related Inflammatory bowel disease phenomenon

**DOI:** 10.1093/rap/rkac066.021

**Published:** 2022-09-28

**Authors:** Sultan Iqbal, Abdul Basit

**Affiliations:** Croydon University Hospital, Croydon, United Kingdom; Croydon University Hospital, Croydon, United Kingdom

## Abstract

**Introduction/Background:**

Ankylosing spondylitis is a HLA B27 associated inflammatory spondyloarthropathy. It can be a debilitating disease affecting mobility and quality of life, therefore, effective symptom and disease control is crucial and consists of pharmacological, including biologics, and non-pharmacological modalities. Until recently, pharmacological treatment options for spinal inflammatory disease control was limited to mainly NSAIDs. Development of biological drugs such as TNF inhibitors and selective Interleukin-17 inhibitors have revolutionized the management especially for patients refractory to conventional treatment. However, use of these novel biologics can come at a cost of side effects including risk of infection and exacerbation of underlying inflammatory conditions.

**Description/Method:**

This 60-year-old Afro-Caribbean female was referred to Rheumatology department with 1-year history of non-radiating lower back pain, alternating buttock pain and morning stiffness. On spinal and sacral MRI, she was found to have multiple thoracic and lumbar spine Romanus lesions and active sacroiliitis and had bilateral grade 2-3 sacroiliitis on plain x-rays of the sacroiliac joints. She also had a raised ESR. There was no peripheral joint involvement, psoriasis, inflammatory eye disease or previous history of inflammatory bowel disease. Her HLA-B27 was negative. Subsequently, she was diagnosed with AS. Previously, she had tried NSAIDs via GP including Diclofenac and Naproxen. She was started on Etoricoxib.

Other issues included spinal canal stenosis at L4/L5 due to disc degeneration, facet joint and ligamentum flavum hypertrophy.

For non-pharmacological management she was under the care of physiotherapy team who advised for regular physiotherapy, acupuncture and hydrotherapy.

On follow up, she did not derive benefit from the Etoricoxib (BASDAI 8.5 and VAS 75), consequently underwent pre-biologic work up. After these were confirmed as normal she was started on Secukinumab 150mg monthly injections (after weekly loading) as per the patient’s preferred biologic choice.

After initiating Secukinumab, her AS improved significantly (reductions in BASDAI to 2.5 and VAS 30). However, after six months, she developed gastrointestinal symptoms with mainly bloating, intermittent diarrhoea and epigastric abdominal pain. She then developed gradual unintentional weight loss.

Under gastroenterologist care, she had extensive diagnostic workup including upper GI endoscopy and colonoscopy (with biopsies), CT-TAP, MRI small bowel and faecal elastase, all of which were normal. However, her faecal calprotectin was high at 130mg/kg (normal range 0-51mg/kg).

Secukinumab was continued given her spinal disease improvement, however, after 18 months of therapy and ongoing gastrointestinal symptoms, Secukinumab was stopped in May-2021. Within weeks of stopping her GI symptoms completely resolved.

**Discussion/Results:**

Use of Secukinumab has been licensed for rheumatological diseases such as psoriatic arthritis, AS and plaque psoriasis. It works by antagonizing interleukin 17, which is a pro-inflammatory cytokine. We started Secukinumab in this patient as per patient choice.

Considering she developed gastrointestinal symptoms it was important to rule out IBD as Secukinumab has been associated with exacerbating underlying IBD or even inducing new cases. For this purpose, part of our workup included faecal calprotectin, colonoscopy and MRI to rule out IBD.

We recommend for patient who are being treated with Secukinumab that if they develop gastrointestinal symptoms it is important to consider iatrogenic IBD. It is recommended in such cases to discontinue Secukinumab and consider alternative biologics with a different mode of action.

It is still not clear what risk factors may help to predict which patients treated with Secukinumab for AS (or other seronegative disorders) could develop iatrogenic IBD. It is also unclear why the epiphenomenon of IBD can be exacerbated by IL17 inhibition when it is clear that such cytokine targeting in AS, psoriasis and psoriatic arthritis is evidently beneficial. Is there a role in pre-biologic screening and monitoring during treatment using faecal calprotectin?

**Key learning points/Conclusion:**

Though Secukinumab is an effective biologic in the management of symptoms and disease control in Ankylosing spondylitis it can be associated with either flare or development of Inflammatory Bowel Disease phenomenon.

